# Supplementary material for: Economic Burden of Pneumococcal Disease in Individuals Aged 15 Years and Older in the Liguria Region of Italy
Source: Vaccines (Basel). 2021 Nov 24;9(12):1380. doi: 10.3390/vaccines9121380 (PMC8706914; doi:10.3390/vaccines9121380)
Supplement: Supplementary file 1 [file vaccines-09-01380-s001.zip › vaccines-1448733-supplementary.pdf]

Supplemental Table S1. Search terms and ICD-9 codes of pneumococcal disease.

Manifestation		ICD-9-CM Code
Pneumonia	Specific	481<br>482.9 and 041.2<br>485 and 041.2<br>486 and 041.2
	Unspecified	482.9<br>485<br>486
	All-cause	480.x<br>481<br>482.x<br>483.x<br>484.x<br>485<br>486<br>487.0
Meningitis	Specific	320.1<br>320.2 and 041.2<br>320.82/320.9 and 041.2<br>322.9 and 041.2
	Unspecified	320.2<br>320.82/320.9
Bacteremia	Specific	038.2<br>038.0 and 041.2<br>038.9 and 041.2<br>790.7 and 041.2
	Unspecified	038.0<br>038.9<br>790.7
AOM		381.x<br>382.x<br>384.0

AOM: acute otitis media; ICD-9-CM: International Classification of Diseases, Ninth Revision, Clinical Modification.
